# Diagnostic value of CT radiomics and clinical features in differentiating focal organizing pneumonia from peripheral lung cancer

**DOI:** 10.3389/fonc.2025.1620217

**Published:** 2025-06-25

**Authors:** Weihua Tang, Huadong Chen, Peijun Liu, Yunxuan Zhang

**Affiliations:** ^1^ Graduate Training Base, Jinzhou Medical University (Renmin Hospital of Wuhan University), Wuhan, Hubei, China; ^2^ Department of Radiology, The Central Hospital of Enshi Tujia and Miao Autonomous Prefecture, Enshi, China; ^3^ Department of Radiology, The Second Hospital of Huangshi, Huangshi, China

**Keywords:** computed tomography, radiomics, focal organizing pneumonia, peripheral lung cancer, clinical features

## Abstract

**Objective:**

This study aimed to evaluate the diagnostic value of computed tomography (CT) radiomics combined with clinical characteristics in differentiating focal organizing pneumonia (FOP) from peripheral lung cancer (PLC).

**Methods:**

A total of 60 FOP patients admitted between June 2023 and June 2024 were included as the FOP group, while 60 PLC patients were assigned to the PLC group. General clinical and imaging data were collected for both groups. Logistic regression analysis was employed to identify independent risk factors for FOP. Radiomics features were extracted from CT images of FOP patients, and the Lasso method was used to select key radiomics features and calculate CT radiomics scores. The diagnostic performance of CT radiomics and clinical characteristics for FOP was assessed using receiver operating characteristic (ROC) curve analysis.

**Results:**

There were no statistically significant differences in age, gender, lung tissue boundary, pleural indentation sign, vascular convergence sign, pleural traction sign, or bronchial air sign between the FOP and PLC groups (*P* > 0.05). However, significant differences were observed in pleural adhesion, lesion location in the outer lung zone, liquefaction necrosis, cavity formation, and spiculation (*P* < 0.05). Logistic regression analysis identified pleural adhesion, lesion location in the outer lung zone, liquefaction necrosis, cavity formation, and long spiculation as independent risk factors for FOP (*P* < 0.05). ROC curve analysis demonstrated that the area under the curve (AUC) for clinical characteristics and CT radiomics in diagnosing FOP were 0.895 and 0.859, respectively. Notably, the AUC for the combined model integrating CT radiomics and clinical characteristics was 0.955, which was significantly higher than that of either approach alone (*P* < 0.05).

**Conclusion:**

Pleural adhesion, lesion location in the outer lung zone, liquefaction necrosis, cavity formation, and long spiculation are key risk factors for FOP. Both CT radiomics and clinical characteristics can aid in the differentiation of FOP from PLC, and their combination significantly enhances diagnostic accuracy.

## 1 Introduction

Peripheral lung cancer (PLC) is a malignant tumor originating from alveoli or small bronchi. In its early stages, PLC may present with no obvious symptoms. However, as the disease progresses, patients may develop dyspnea, chest pain, and coughing (1, 2). Organizing pneumonia (OP) is an interstitial lung disease defined by clinical, histological, and radiological features. Its histopathological characteristics include granulation tissue deposition in alveolar ducts and alveoli, with varying degrees of terminal bronchiole involvement, often forming characteristic Masson bodies (3, 4). Focal organizing pneumonia (FOP) is a rare manifestation of OP, classified as an infectious pulmonary lesion (5). Its pathological features include fibroblast proliferation and interstitial fibrotic tissue forming granulation tissue that fills the alveolar spaces with associated inflammatory infiltration (6). Unlike PLC, FOP is a benign lesion. Radiologically, FOP often appears as a pulmonary mass or solitary pulmonary nodule. Clinically, FOP has nonspecific symptoms, with most patients presenting only with mild cough or being asymptomatic in the early stages (7). In cases where the lesion is extensive, patients may experience exertional dyspnea and ventilation dysfunction.

Although FOP and PLC have distinct pathological characteristics, their clinical symptoms and physical signs lack specific differentiation. The imaging features of FOP are highly variable, making it difficult to distinguish from PLC. Consequently, FOP is often misdiagnosed as PLC, leading to unnecessary fine-needle aspiration biopsies or even lobectomy in affected patients (8, 9). Therefore, there is an urgent need for a highly accurate and non-invasive diagnostic approach to differentiate FOP from PLC, thereby reducing unnecessary invasive procedures.

Radiomics, a high-throughput and non-invasive analytical approach, extracts quantitative imaging features from standard medical images, including shape descriptors, texture parameters, and intensity-based metrics. This technique allows for objective and precise characterization of lesions (10, 11). This study aims to evaluate the diagnostic value of CT-based radiomics combined with clinical characteristics in distinguishing FOP from PLC, providing valuable insights for improving the accuracy of FOP and PLC diagnosis.

## 2 Methods

### 2.1 Study population

This retrospective study enrolled all consecutive patients admitted to our institution between June 1, 2023 and June 30, 2024, including 60 cases of focal organizing pneumonia (FOP) and 60 cases of peripheral lung cancer (PLC). Inclusion criteria for the FOP group were: age ≥18 years; diagnosis confirmed by CT-guided percutaneous lung biopsy or surgical resection; first-time onset without prior treatment before admission; and provision of written informed consent. Exclusion criteria included: psychiatric disorders or impaired consciousness that could interfere with study participation; pregnancy or lactation; coexisting cardiopulmonary diseases that could affect CT interpretation; or poor-quality CT images. For the PLC group, inclusion criteria were: age ≥18 years; histopathological confirmation of PLC; absence of intrapulmonary or distant metastases; first-time onset without prior treatment; and informed consent provided. Exclusion criteria were consistent with the FOP group and additionally included a history of other malignancies. This study was approved by the Ethics Committee of The Central Hospital of Enshi Tujia and Miao Autonomous Prefecture (approval number: 2025-089-01). All patient data were carefully anonymized and handled in strict accordance with data protection regulations to ensure the privacy and confidentiality of the participants throughout the study.

### 2.2 CT imaging examination

A GE Optima CT520 Pro scanner was used to perform CT scans on both groups of patients, with the scanning range extending from the lung apex to the costophrenic angle. The scanning parameters included a tube voltage of 120 kVp, a tube current of 250 mA, a slice thickness of 5 mm, and a slice interval of 5 mm. Iodixanol (mgI/mL) was used as the contrast agent, with 80–90 mL injected at a rate of 2.5–3.0 mL/s. Arterial phase and venous phase scans were performed at 25–30 seconds and 55–60 seconds after contrast injection, respectively. The CT images were exported via the image archiving and communication system. Two experienced radiologists independently evaluated the image quality. In case of discrepancies, consensus was reached through discussion.

### 2.3 Image segmentation and feature extraction

The CT images were imported into LIFEx software. Manual segmentation was performed by two radiologists with 10 and 15 years of experience in radiological diagnosis. The radiologist with 10 years of experience delineated the region of interest (ROI) and extracted features twice, with a two-week interval. The radiologist with 15 years of experience performed a single ROI delineation and feature extraction. First, the CT images were imported into specialized imaging analysis software. Both radiologists manually delineated the regions of interest covering the entire lesion area while avoiding adjacent normal tissues. To assess reproducibility, the radiologist repeated the ROI delineation after a two-week interval. The intraclass correlation coefficient (ICC) was calculated to evaluate the consistency and stability between the two radiologists as well as the intra-observer reproducibility. Any discrepancies were resolved through discussion to ensure the accuracy and reliability of the ROI delineation. The CT images and corresponding ROIs were resampled to a voxel size of 1 mm × 1 mm × 1 mm using Analysis-Kit software, which extracted 1,826 radiomic features from the CT images. The ICC was used to evaluate the consistency of the radiomic features. After ICC filtering, 22 stable radiomic features were retained for further analysis.

### 2.4 Statistical analysis

All statistical analyses were performed using SPSS version 24.0. The t-test was used to compare continuous variables that conformed to a normal distribution, and the chi-square test was used to compare categorical variables. A p-value of <0.05 was considered statistically significant. Logistic regression was used to identify risk factors for FOP. Lasso regression was applied to select significant radiomic features. To reduce overfitting during feature selection and model development, we employed a 5-fold cross-validation strategy to ensure model robustness and avoid overfitting to the training data. In addition, regularization methods and strict feature selection were applied to reduce model complexity. A post‐hoc power analysis was conducted using the observed difference in AUC between the radiomics and clinical models (effect size d = 0.15). Feature importance was assessed by ranking the absolute values of the LASSO regression coefficients, and SHAP values were computed to quantify each feature’s contribution to the combined model’s predictions. This dual approach enhances model interpretability by revealing which clinical and radiomic features drive diagnostic decisions. Receiver operating characteristic (ROC) curves based on CT radiomics and clinical features were generated using MedCalc software to evaluate diagnostic performance for FOP. All statistical analyses were performed using SPSS version 24.0. The t-test was used to compare continuous variables that conformed to a normal distribution, and the chi-square test was used to compare categorical variables. A p-value of <0.05 was considered statistically significant. Logistic regression was used to identify risk factors for FOP. Lasso regression was applied to select significant radiomic features. ROC curves based on CT radiomics and clinical features were generated using MedCalc software to evaluate diagnostic performance for FOP.

## 3 Result

### 3.1 Analysis of general characteristics between the two groups

**Figure 1:**
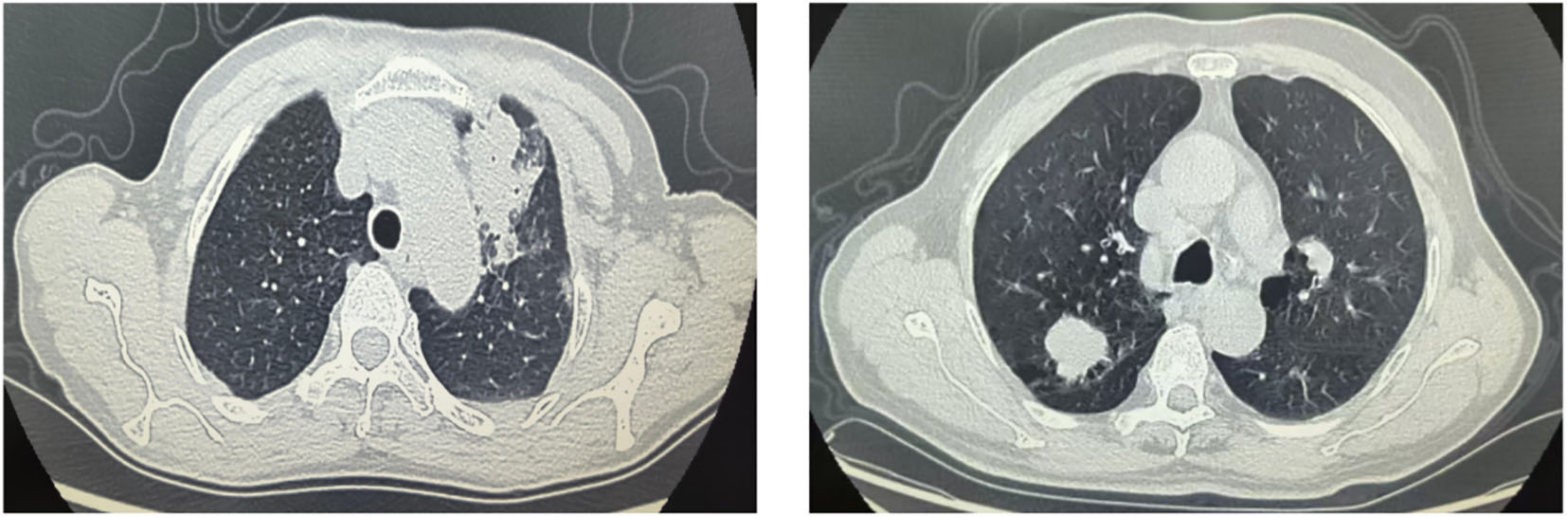
Distinct imaging features of FOP and PLC.

There were no statistically significant differences between the FOP and PLC groups in terms of age, sex, lesion-lung interface clarity, pleural indentation sign, vessel convergence sign, pleural retraction sign, or air bronchogram (*P* > 0.05). However, significant differences were observed in features such as lesions abutting the pleura, lesions located in the peripheral lung zone, liquefactive necrosis, cavitation, and spiculation (*P* < 0.05). Detailed information is provided in **Table 1** and **Figure 1**.

**Table 1:** General characteristics of patients in the FOP and PLC groups.

Variable	FOP group (n=60)	PLC group (n=60)	t/χ²	P-value
Age (years)	56.82 ± 6.59	57.24 ± 7.13	0.335	0.738
Sex			0.301	0.581
Male	32 (53.33%)	35 (58.33%)		
Female	28 (46.67%)	25 (41.67%)		
Adjacent to pleura			19.221	0.000
Yes	41 (68.33%)	17 (28.33%)		
No	19 (31.67%)	43 (71.67%)		
Boundary with lung parenchyma			0.139	0.709
Clear	23 (38.33%)	25 (41.67%)		
Blurred	37 (61.67%)	35 (58.33%)		
Peripheral zone location			11.627	0.001
Yes	31 (51.67%)	13 (21.67%)		
No	29 (48.33%)	47 (78.33%)		
Pleural indentation			0.301	0.583
Present	33 (55.00%)	30 (50.00%)		
Absent	27 (45.00%)	30 (50.00%)		
Vascular convergence			0.342	0.559
Present	18 (30.00%)	21 (35.00%)		
Absent	42 (70.00%)	39 (65.00%)		
Lobulation			1.443	0.210
Present	47 (78.33%)	52 (86.67%)		
Absent	13 (21.67%)	8 (13.33%)		
Pleural traction			0.304	0.581
Present	25 (41.67%)	28 (46.67%)		
Absent	35 (58.33%)	32 (53.33%)		
Air bronchogram			0.534	0.465
Present	27 (45.00%)	31 (51.67%)		
Absent	33 (55.00%)	29 (48.33%)		
Liquefactive necrosis			14.737	0.000
Yes	39 (65.00%)	18 (30.00%)		
No	21 (35.00%)	42 (70.00%)		
Cavitation		14.903	0.000
Yes	37 (67.67%)	16 (26.67%)		
No	23 (38.33%)	44 (73.33%)		
Spiculated margin			18.502	0.000
Long spiculation	35 (58.33%)	12 (20.00%)		
None or short spiculation	25 (41.67%)	48 (80.00%)		

### 3.2 Risk factors for FOP

A multivariate logistic regression analysis was conducted to explore the potential risk factors associated with FOP. Variables considered as independent predictors included: subpleural location of the lesion, lesion situated in the peripheral zone of the lung, presence of liquefactive necrosis, cavitation, and long spiculation. The presence of FOP was used as the dependent variable. The specific variable assignments for the analysis are detailed in **Table 2**.

**Table 2:** Variable assignment table.

Variable	Assignment Method
FOP	PLC group = “0”; FOP group = “1”
Subpleural Location	No = “0”; Yes = “1”
Lesion in Peripheral Zone	No = “0”; Yes = “1”
Liquefactive Necrosis	No = “0”; Yes = “1”
Cavitation	No = “0”; Yes = “1”
Spiculation	No spiculation or short spiculation = “0”; Long spiculation = “1”

The logistic regression results revealed that all the aforementioned imaging features—subpleural lesion attachment, peripheral lesion location, liquefactive necrosis, cavitary changes, and long spiculated margins—were significantly associated with the diagnosis of FOP, with *P*-values less than 0.05, indicating their role as independent risk factors. These findings suggest that these radiological characteristics may provide important diagnostic clues for differentiating FOP from other pulmonary pathologies (**Table 3**).

**Table 3:** Logistic regression analysis of FOP.

Factor	Regression Coefficient	Standard Error	Wald Value	P Value	OR	95% Confidence Interval (CI)
Subpleural Location	2.114	0.550	14.788	0.000	8.283	2.820 – 24.333
Lesion in Peripheral Zone	1.768	0.567	9.734	0.002	5.862	1.930 – 17.804
Liquefactive Necrosis	1.674	0.528	10.041	0.002	5.336	1.894 – 15.032
Cavitation	1.829	0.549	11.119	0.001	6.229	2.126 – 18.256
Long Spiculation	1.500	0.543	7.645	0.006	4.482	1.548 – 12.979
Constant	-3.783	0.702	29.048	0.000	0.023	—

### 3.3 Feature selection results

**Figure 2:**
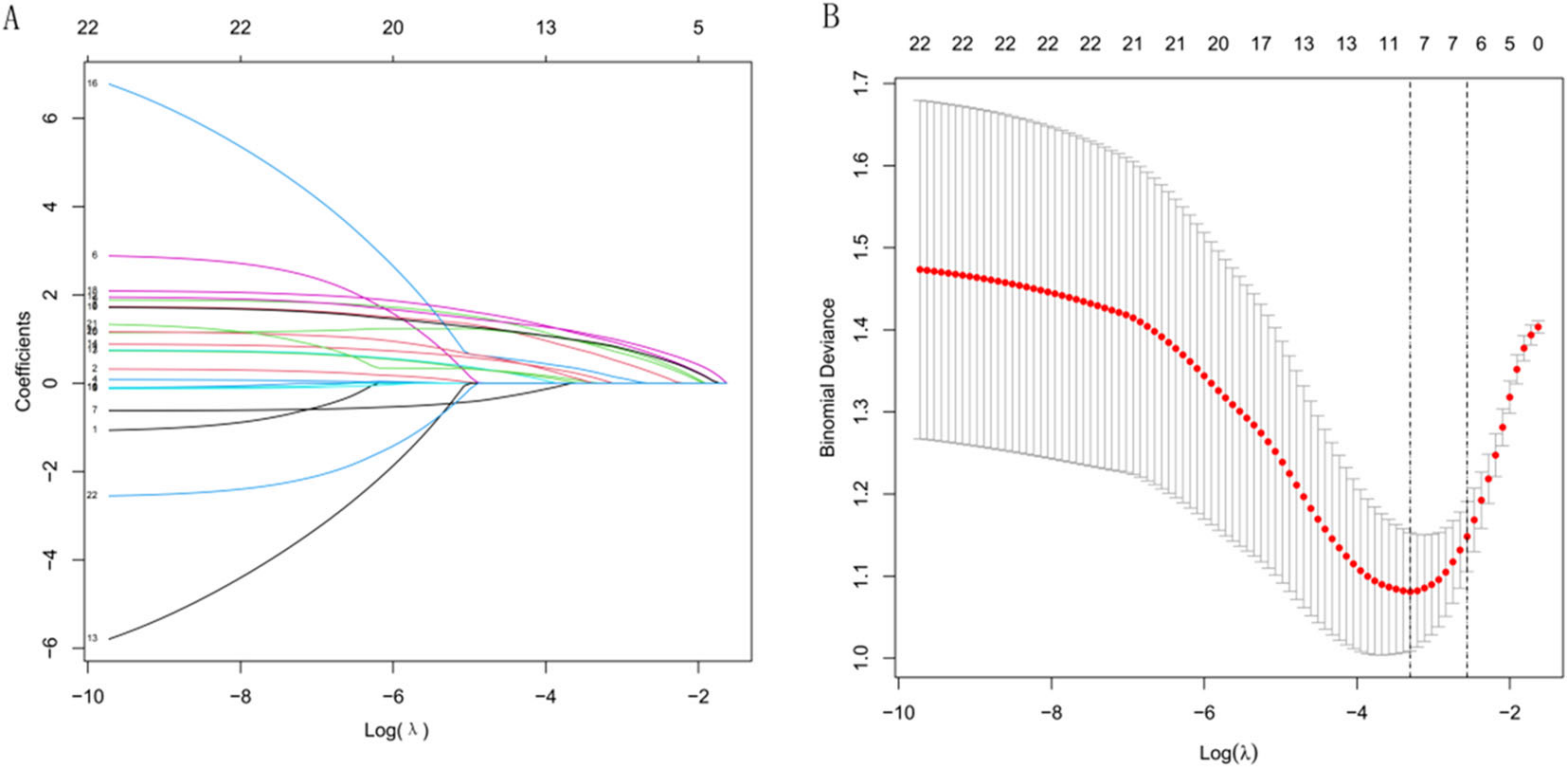
Radiomics feature selection using LASSO regression. **(A)** Coefficient profiles of variables under different values of log λ. **(B)** Selection of the optimal λ based on binomial deviance.

Lasso regression analysis was performed on the 22 extracted radiomic features, resulting in the selection of six features with non-zero coefficients, indicating their importance in distinguishing between groups (**Figure 2**). These selected features were subsequently used to construct a radiomics score through a weighted linear combination of their respective coefficients. The final formula for the radiomics score is as follows: Radiomics Score = 0.18206 × wavelet_LHL_glszm_ZonePercentage + 0.17458 × logarithm_firstorder_Range + 0.21084 × original_shape_Sphericity + 0.16917 × lbp_3D_m2_glszm_SizeZoneNonUniformity + 0.12852 × lbp_3D_k_glszm_ZoneEntropy + 0.18632 × wavelet_HHH_ngtdm_Coarseness − 0.05869. This equation represents a comprehensive radiomic signature that integrates key imaging features with potential diagnostic value.

### 3.4 Diagnostic performance of CT-based radiomics and clinical features for differentiating FOP

**Figure 3:**
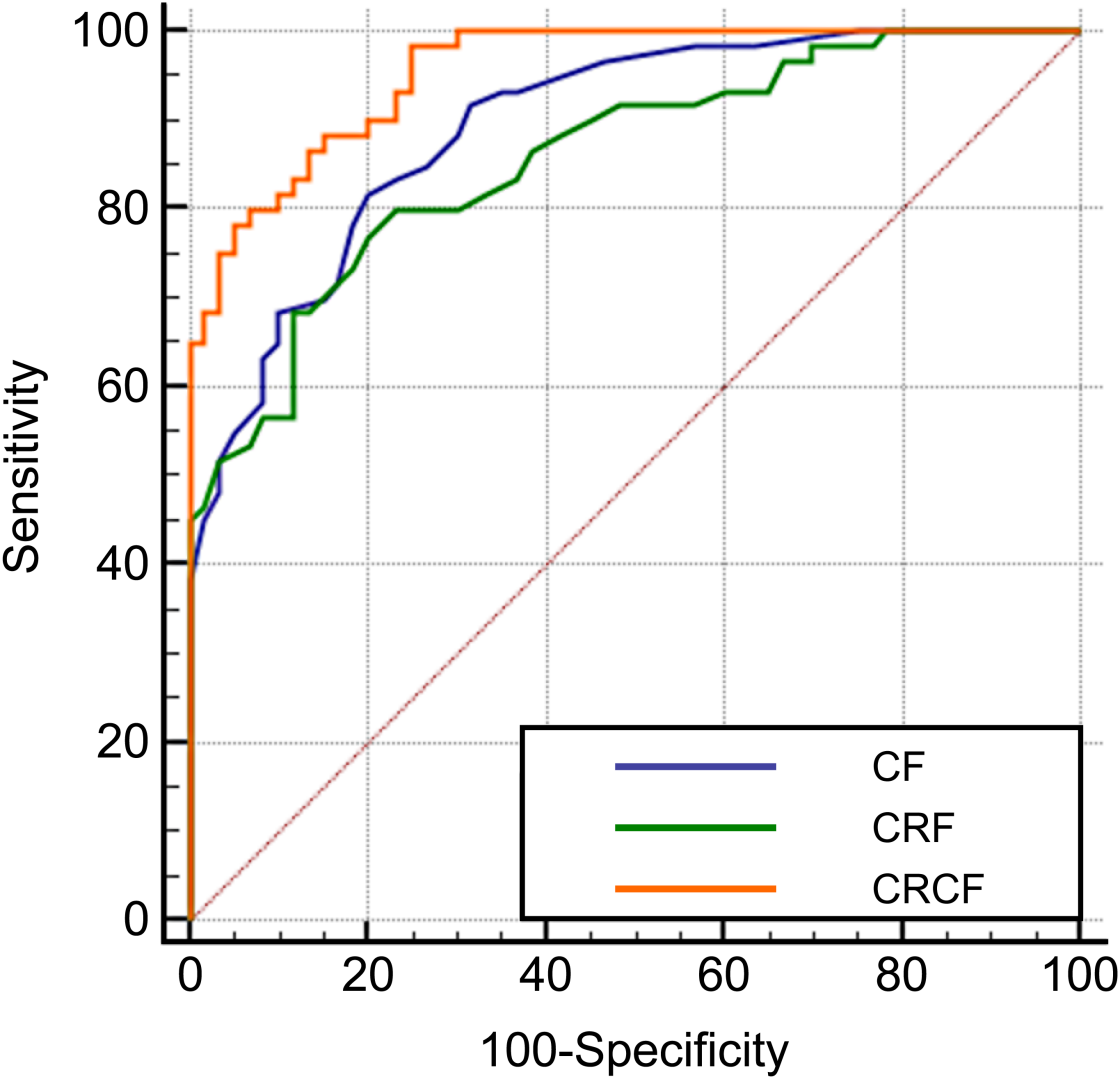
ROC curves for the diagnosis of FOP using CT radiomics and clinical features. Receiver operating characteristic (ROC) curves illustrating the diagnostic performance of Clinical Features (CF), CT Radiomics Features (CRF), and the Combined Radiomics and Clinical Features (CRCF) model in distinguishing FOP from PLC.

ROC curve analysis was performed to evaluate the diagnostic efficacy of clinical features, radiomics features, and their combination for identifying focal organizing pneumonia (FOP). The area under the ROC curve (AUC) for clinical features alone was 0.895, while the AUC for radiomics features alone was 0.859. When radiomics features were integrated with clinical variables, the diagnostic performance improved substantially, with a combined AUC of 0.955. Statistical analysis indicated that the combined model significantly outperformed the individual models based on either clinical or radiomics features alone (*P* < 0.05). These findings highlight the added value of combining CT-derived radiomics with clinical data in enhancing diagnostic accuracy for FOP. Detailed results are provided in **Table 4** and **Figure 3**. A *post hoc* power analysis was performed to assess whether the sample size was sufficient to detect a statistically meaningful difference between the diagnostic models. Based on the observed difference in AUC between the radiomics and clinical models, an effect size of d = 0.15 was estimated. With a significance level of α = 0.05 and two-sided testing, the current total sample size of 120 patients (60 per group) yields approximately 82% power to detect this difference, suggesting that the study was adequately powered for this comparison.

**Table 4:** Diagnostic performance of CT-based radiomics and clinical features for FOP.

Predictor	AUC	Standard Error	P-value	Sensitivity	Specificity	95% CI
Clinical Features	0.895	0.027	0.000	81.70%	80.00%	0.826–0.944
CT Radiomics Features	0.859	0.033	0.000	80.00%	76.70%	0.783–0.915
Combined Radiomics and Clinical Features	0.955	0.015	0.000	98.30%	75.00%	0.901–0.985

## 4 Discussion

FOP is a localized inflammatory lesion of lung tissue caused by viruses or bacteria and is considered a subtype of organizing pneumonia. It predominantly occurs in middle-aged and elderly individuals, often without obvious clinical symptoms, and is mostly detected during routine physical examinations (12, 13). The pathological changes of FOP mainly involve fibroblast proliferation along the alveolar walls, which gradually extends to the alveolar ducts and alveolar spaces as the disease progresses (13, 14). PLC, on the other hand, originates from the area between the tertiary bronchi and respiratory bronchioles and is a common type of pulmonary malignancy, with adenocarcinoma being the most prevalent histological type (15, 16). FOP and PLC share several similarities, with commonly reported symptoms including dyspnea, low-grade fever, chest pain, hemoptysis, and productive cough. Lesions in both conditions typically present as pulmonary masses or nodules, which often leads to FOP being misdiagnosed as PLC (8, 17). This misdiagnosis may result in invasive procedures such as fine-needle aspiration or lobectomy. At present, FOP responds well to corticosteroid pulse therapy, from which most patients can benefit. However, pathological examination of tissue remains the gold standard for diagnosing FOP, and the process of obtaining such samples may carry certain risks (18, 19). In recent years, radiomics has offered a new approach for disease diagnosis. Radiomics can extract a vast, comprehensive, and in-depth array of features, while minimizing the influence of visual fatigue and subjective experience such as clinical expertise (20, 21).

This study found that the proportion of lesions abutting the pleura was higher in the FOP group compared to the PLC group. Pleural abutment was identified as a risk factor for FOP, consistent with findings reported in previous studies (22). Lesions near the pleura may trigger localized inflammatory responses, leading to interactions between the lung parenchyma and the pleura, thereby promoting the development of FOP. Therefore, when lesions are closely adjacent to the pleura, the possibility of FOP should be strongly considered. Additionally, this study revealed that lesions located in the peripheral zone of the lung were more prevalent in the FOP group than in the PLC group, and peripheral distribution was also found to be a risk factor for FOP. This may be due to the fact that the peripheral lung regions are more susceptible to external environmental influences, such as inhalation of harmful substances or infections, which may induce FOP. Moreover, the peripheral zone may be more involved in the processes of alveolar injury and repair. Since the pathological hallmark of FOP is the formation of fibrous tissue within the alveoli, the development of such fibrotic changes may be associated with peripheral lung lesions.

The results of this study indicate that the proportion of liquefactive necrosis was higher in the FOP group compared to the PLC group, and liquefactive necrosis was identified as a risk factor for FOP. Liquefactive necrosis is typically associated with inflammatory responses, in which the infiltration of inflammatory cells and the release of cytokines may contribute to the development of FOP. In FOP, inflammation induces the formation of granulation tissue within the alveolar ducts and alveoli as part of the body’s repair process; however, this may also disrupt normal tissue architecture. Therefore, liquefactive necrosis may play a key role in the pathogenesis of FOP.

This study also found that the incidence of cavitation was higher in the FOP group than in the PLC group, and cavitation was identified as a risk factor for FOP, which is consistent with findings reported in previous research (8, 23). Cavities are often formed in areas of necrotic lung tissue, which may result from inflammation, infection, or other pathological processes. In FOP, inflammatory responses may cause localized tissue damage or necrosis in the lungs, thereby contributing to cavity formation and linking it to the pathological mechanisms of FOP. Additionally, this study showed that the proportion of lesions with spiculated margins was higher in the FOP group than in the PLC group, and spiculated margins were found to be a risk factor for FOP. Spiculated margins are typically associated with processes of tissue injury and repair. In FOP, inflammation-induced tissue damage may lead to granulation tissue formation, and spiculated margins may represent the radiologic manifestation of this granulation tissue.

At present, radiomics has shown promising potential in the diagnosis of pulmonary diseases. Previous studies have demonstrated that CT-based radiomics models can effectively differentiate organizing pneumonia from pulmonary lymphoma (24, 25). Similarly, other research has shown that CT radiomics models are capable of distinguishing organizing pneumonia from primary mucinous adenocarcinoma of the lung (26). In this study, three models were constructed based on multivariate analysis and feature selection results. The AUC for diagnosing FOP using clinical features alone was 0.895, while the AUC for the CT radiomics model alone was 0.859. When CT radiomics features were combined with clinical features, the diagnostic AUC increased to 0.955. The combined model demonstrated a significantly higher diagnostic performance than either clinical features or radiomics alone (*P* < 0.05). These findings suggest that both CT radiomics and clinical features are useful for distinguishing FOP from PLC, and their combination can further enhance diagnostic accuracy. This study is limited by a relatively small sample size, which may affect the statistical power and generalizability of the findings. Future multicenter and prospective studies with larger cohorts are planned to validate the model across diverse populations and imaging conditions. This study faces several practical challenges, including the lack of standardized imaging protocols, variability across scanners and institutions, the time-consuming nature of manual segmentation, and limited model interpretability. To address these issues, we plan to conduct a prospective pilot implementation and usability study to assess the model’s feasibility, diagnostic value, and integration potential in real-world clinical settings. Nonetheless, our findings provide valuable preliminary evidence supporting the potential of combining clinical and radiomic features for improved diagnosis.

## 5 Conclusion

This study identified pleural abutment, peripheral lesion location, liquefactive necrosis, cavitation, and spiculated margins as significant risk factors for focal FOP. Both CT radiomics and clinical features demonstrated good diagnostic value in distinguishing FOP from PLC. Moreover, the combination of radiomics and clinical data significantly improved diagnostic accuracy, offering a non-invasive and reliable approach for early identification of FOP. These findings provide useful insights that may assist clinicians in making more accurate diagnoses and optimizing treatment strategies.

## Data Availability

The original contributions presented in the study are included in the article/supplementary material. Further inquiries can be directed to the corresponding author.
